# Effects of Water Depth, Seasonal Exposure, and Substrate Orientation on Microbial Bioerosion in the Ionian Sea (Eastern Mediterranean)

**DOI:** 10.1371/journal.pone.0126495

**Published:** 2015-04-20

**Authors:** Claudia Färber, Max Wisshak, Ines Pyko, Nikoleta Bellou, André Freiwald

**Affiliations:** 1 Senckenberg am Meer, Abteilung Meeresforschung, Südstrand 40, 26382 Wilhelmshaven, Germany; 2 GeoZentrum Nordbayern, Fachgruppe Paläoumwelt, Universität Erlangen-Nürnberg, Loewenichstraße 28, 91054 Erlangen, Germany; 3 Hellenic Centre for Marine Research, Institute of Oceanography, 46.7 km Athens Sounio, 19003 Anavyssos, Greece; Universität Göttingen, GERMANY

## Abstract

The effects of water depth, seasonal exposure, and substrate orientation on microbioerosion were studied by means of a settlement experiment deployed in 15, 50, 100, and 250 m water depth south-west of the Peloponnese Peninsula (Greece). At each depth, an experimental platform was exposed for a summer period, a winter period, and about an entire year. On the up- and down-facing side of each platform, substrates were fixed to document the succession of bioerosion traces, and to measure variations in bioerosion and accretion rates. In total, 29 different bioerosion traces were recorded revealing a dominance of microborings produced by phototrophic and organotrophic microendoliths, complemented by few macroborings, attachment scars, and grazing traces. The highest bioerosion activity was recorded in 15 m up-facing substrates in the shallow euphotic zone, largely driven by phototrophic cyanobacteria. Towards the chlorophyte-dominated deep euphotic to dysphotic zones and the organotroph-dominated aphotic zone the intensity of bioerosion and the diversity of bioerosion traces strongly decreased. During summer the activity of phototrophs was higher than during winter, which was likely stimulated by enhanced light availability due to more hours of daylight and increased irradiance angles. Stable water column stratification and a resulting nutrient depletion in shallow water led to lower turbidity levels and caused a shift in the photic zonation that was reflected by more phototrophs being active at greater depth. With respect to the subordinate bioerosion activity of organotrophs, fluctuations in temperature and the trophic regime were assumed to be the main seasonal controls. The observed patterns in overall bioeroder distribution and abundance were mirrored by the calculated carbonate budget with bioerosion rates exceeding carbonate accretion rates in shallow water and distinctly higher bioerosion rates at all depths during summer. These findings highlight the relevance of bioerosion and accretion for the carbonate budget of the Ionian Sea.

## Introduction

Biological erosion of carbonate substrate is a key process during the (re)cycling of calcium carbonate and the formation of calcareous sediments in the ocean [1, 2]. Wherever fresh substrate becomes available it is rapidly colonised by pioneer microbial euendoliths, finding shelter from grazers and physical disturbance or seeking nutrition within the substrate [3–5]. Mature microboring communities usually develop during the first 6 months of exposure [6, 7], while macroborers and grazers are considered to take more than 2–3 years to fully establish [8]. In order to investigate this succession and the effect of ecological conditions on bioerosion, intensive research has been carried out in the past years focusing mainly on tropical coral reef ecosystems [9–12]. However, from bioerosion experiments conducted in the non-polar Atlantic Ocean it is known that bioerosion follows a distinct latitudinal gradient with highest intensities in the tropics [13–15], intermediate levels in the warm-temperate [16, 17], and lowest rates in the cold-temperate high latitudes [18]. In modern oceans, many euendoliths are considered to show a cosmopolitan distribution (i.e. they occur worldwide in suitable habitats). For the bathymetric zonation of phototrophic euendoliths and the bioerosion trace assemblages they produce (ichnocoenoses), light was identified as main controlling factor [15, 19–21]. In contrast, endolithic marine fungi are organotrophs biodegrading calcareous shells in order to exploit mineralised organic matter and are independent of light [22]. The knowledge on the effects of water temperature, salinity, inorganic nutrients, particulate organic matter, and the complex interplay of all these factors on microbial bioerosion is still limited [23].

In the Mediterranean Sea, ongoing eutrophication, pollution, warming, and ocean acidification are considered to magnify the intensity of bioerosion [24]. In contrast to tropical settings, the main bioerosion agents are considered to be microendolithic cyanobacteria, chlorophytes, and fungi, alongside macroboring sponges, molluscs, and grazing sea urchins, while fish apparently do not play a significant role [24]. Taxonomical studies on bioeroding cyanobacteria and chlorophytes have been carried out in the Mediterranean Sea since the late 19^th^ century [25–29]. But experimental studies remain scarce and were mainly concentrated on the Marseilles region in the Western Mediterranean Sea [30, 31]. From the sole bioerosion experiment conducted in the Eastern Mediterranean Sea, only preliminary results are presently published [32].

The present study was part of a carbonate cycling experiment aiming to provide a budget of carbonate bioerosion versus accretion in the Ionian Sea. The Eastern Mediterranean Sea shows a high spatio-temporal variability with respect to water temperatures, salinity, nutrients, and primary productivity [33, 34]. This provides prime conditions in order to investigate the effects of water depth, seasonal exposure, and substrate orientation on bioerosion and accretion. The experiment comprised 12 platforms with experimental substrates deployed along a bathymetric transect in 15, 50, 100, and 250 m. Per depth one platform was exposed for a summer period (February-October), a winter period (October-April), and about an entire year. In this paper, we report on the analysis of the effects of water depth, seasonal exposure, and substrate orientation on bioerosion rates and traces, and discuss our results in context of the spatio-temporal environmental variability of the Ionian Sea.

## Material and Methods

### Ethics statement

The Hellenic Centre for Marine Research (HCMR) was consortium partner in the projects KM3NET (Contract No. 011937; EU-FP6), POSEIDON II (EFTA & Greek Ministry of National Economy) and POSEIDON III (Contract no. EL0048; EEA Grants) under which the cruises for this study were performed.

The HCMR is a governmental research institute and belongs to the Ministry of Education and Religious affairs. Hence, it has the permit to obtain scientific fieldwork in Greek Seas and along the coastline and was represented during the cruises by coauthor Nikoleta Bellou. Prior to the cruises the local authorities were informed about the planned experiment and the coordinates of the deployed experiment were provided. As no molecular biological analyses are planned to be performed no further permit and approval were needed.

### Study site

**Fig 1:**
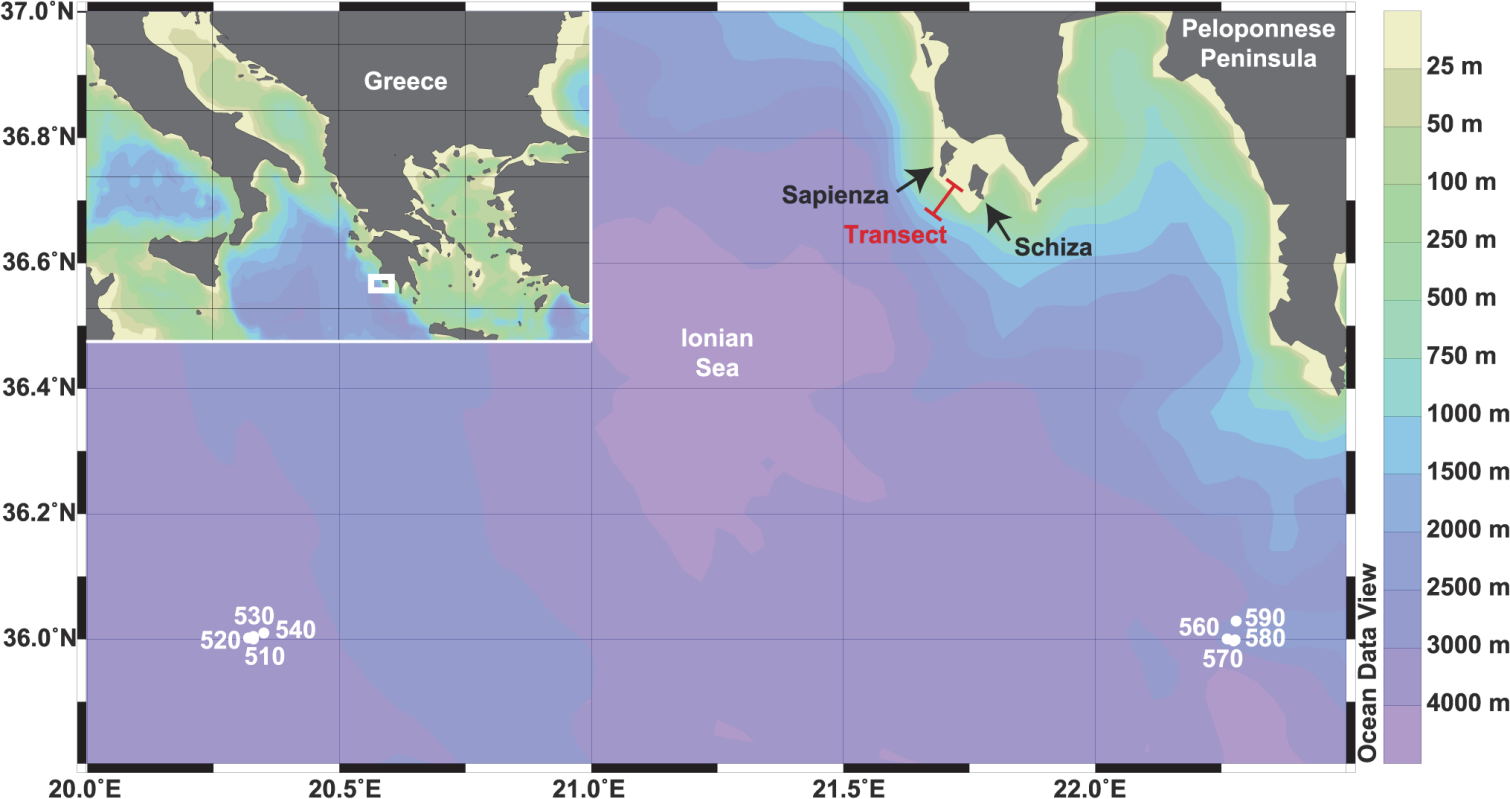
Location of the bathymetric transect (15, 50, 100, and 250 m) south-west of the Peloponnese Peninsula in the Ionian Sea. Numbers show the locations of light measurements that were carried out in the framework of the European Commission’s Marine Sciences & Technology (MAST) program [38] (S1 Fig). Base map displayed in Ocean Data View [39].

The carbonate cycling experiment was carried out south-west of the Peloponnese Peninsula (Greece) in the Ionian Sea (ca. 36.73° N, 21.68° E, Fig 1). The area is far from any river discharge and characterised by exceptionally clear and oligotrophic water with low sedimentation rates [35, 36]. The general structure of water masses in the area is composed of Modified Atlantic Water that shows a subsurface minimum of salinity between 30–200 m water depth and Levantine Intermediate Water with a salinity maximum in 200–600 m [37]. During summer, Ionian Surface Water clearly differs from Modified Atlantic Water by being saltier and warmer [37]. Values of surface irradiance calculated from light measurements of photosynthetically active radiation in the Ionian Sea that were carried out in the framework of the European Commission’s Marine Sciences & Technology (MAST) program [38], suggest an approximate base of the euphotic and dysphotic zones in ca. 100 m and 180 m, respectively (S1 Fig).

### Experimental setup

The deployment of the summer and one year platforms was carried out onboard R/V Aegaeo on 28 February 2008. The summer platforms were recovered and replaced by the winter platforms after 226 days onboard R/V Philia on 11/12 October 2008. The winter and one year platforms were finally retrieved after 173 and 400 days, respectively, onboard R/V Philia on 2 April 2009. The 15 m platforms were deployed by scuba divers, the deeper platforms via remotely operated vehicle. Platforms in 15 m were deployed on rocky hard ground, the deeper platforms on sandy-muddy soft ground. After the winter, the 15 m platform was found turned over and was therefore excluded from analysis.

**Fig 2:**
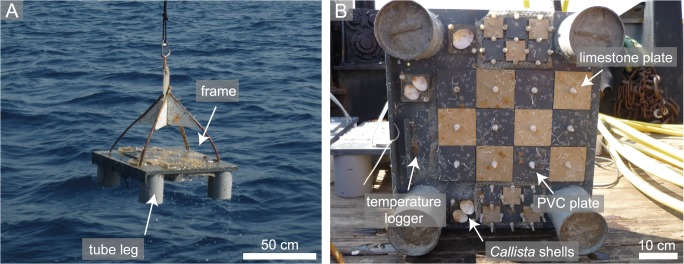
Photographs of an experimental platform. (A) Each platform is composed of a 54 x 60 cm PVC frame with four concrete filled PVC tube legs and experimental substrates on the up- and down-facing side. (B) View of the down-facing side of a platform equipped with limestone and PVC plates for the investigation of bioerosion and accretion rates, shells of the Mediterranean bivalve *Callista chione* for the analysis of bioerosion traces, and a water temperature data logger. Smaller PVC and limestone plates are mounted for the investigation of bacterial biofilms, which are not the scope of the present paper.

The principle design of the platforms is based on previous experiments in the Swedish Kosterfjord [18] and the Azores Archipelago [16, 17, 40]. Each platform is composed of a 54 x 60 cm PVC frame with four concrete filled PVC tube legs and experimental substrates on the up- and down-facing side (Fig 2). In each orientation, six limestone and six PVC plates (10 x 10 x 1 cm) were mounted for the assessment of carbonate bioerosion and accretion rates, as well as three shells of the Mediterranean bivalve *Callista chione*, embedded in a socket of epoxy resin, in order to expose solely the pristine inner side of the valve, for the investigation of bioerosion traces. Smaller PVC and limestone plates were mounted for the investigation of bacterial biofilms, which are not the scope of the present paper. On the down-facing side of each platform, a temperature data logger (Hobo Water Temp Pro v2, accuracy ±0.2°C or Star Oddi Starmon-Mini, accuracy ±0.05°C; 30 min. intervals) was fixed in order to characterise the temperature variability and stratification of water masses during the experiment. Due to data loss, no water temperature measurements are available from the 250 m winter deployment as well as from the entire one year deployment.

### Carbonate budget

Carbonate bioerosion and accretion rates were calculated via the difference in the weight of substrates before and after exposure. For this purpose, limestone and PVC plates were dried before deployment for two weeks at 70°C and weighed with a precision scale (limestone plates: Mettler Toledo Classic Plus PB3002-S, accuracy 10 mg; PVC plates: Mettler Toledo Classic AB 204-S, accuracy 0.1 mg). After retrieval, all non-calcareous components were removed in 15% hydrogen peroxide solution (H_2_O_2_). Then the plates were again dried at 70°C. From limestone blocks, all calcareous components were carefully removed with a scalpel under the microscope. Bioerosion rates were calculated from the weight difference of limestone plates before deployment and after removing calcareous components. Accretion rates were calculated from the difference of the PVC plates before and after deployment, as well as from the weight of calcareous components, which were removed from the limestone plates. Net limestone erosion rates were calculated from the difference of carbonate accretion and bioerosion rates. All rates are given in gram per square metre per year (g m^-2^ yr^-1^).

### Bioerosion traces

Bioerosion traces in *Callista* shells were analysed by scanning electron microscopy (SEM). For this purpose, two shells each from the up- and down-facing side of each platform were selected. The third was stored in 4% formaldehyde solution. For the preparation of epoxy resin casts of bioerosion traces, the shells were treated with H_2_O_2_ for a complete removal of fleshy epiliths, air dried, and casted in epoxy resin (Araldit BY 158, Aradur 21) in a vacuum chamber (Struers CitoVac). From each shell, four surface samples and two cross-sections were cut with a rock saw and decalcified in 5% hydrochloric acid (cross-sections only partially). The epoxy resin blocks, now exhibiting the positive casts of the microborings, were sputter-coated with gold, and analysed under the SEM.

Bioerosion traces on the surface of the limestone plates were documented with a digital light microscope (Keyence VHX-1000D).

Identification of bioerosion traces followed descriptions in [17, 41–43]. The bioerosion traces were ichnotaxonomically identified to ichnospecies level or described in informal nomenclature (e.g. ‘Super thin form’) and semiquantified in the abundance classes ‘very common’, ‘common’, ‘rare’, and ‘very rare’. Due to the removal of carbonate encrusters, the documentation of traces from limestone plates was potentially biased and thus excluded from statistical analyses, albeit listed in the bioerosion inventory.

### Statistical analyses

Due to the loss of the 15 m winter platform, overall tests of the effects of water depth, seasonal exposure, substrate orientation, and substrate type (the latter solely for accretion) on carbonate bioerosion and accretion along the complete transect were not feasible. Instead, 15 m rates were excluded from analysis and the tests only carried out for summer, winter, and one year bioerosion, accretion, and net erosion rates from 50–250 m. Prior to analysis, normality was tested and rejected (p < 0.05) by Shapiro-Wilk tests. Bioerosion and accretion rates were log(x+1) transformed and net limestone erosion rates log(x+10) transformed. The effects of depth, exposure, and orientation on bioerosion and net limestone erosion rates were tested by three-way permutational analysis of variance (PERMANOVA). The effects of depth, exposure, orientation, and substrate type on accretion rates were tested by four-way PERMANOVA. All PERMANOVA tests were based on a fixed factor design and calculated under 999 permutations.

According to the procedure described in [17], semiquantitative abundance data of bioerosion traces was translated into ranked abundance classes where ‘very common’ was transformed to an abundance value of 1000, ‘common’ to 100, ‘rare‘ to 10, and ‘very rare‘ to 1. In order to identify similarities among bioerosion traces multidimensional scaling analysis (MDS), in combination with a group-average cluster analysis based on Bray-Curtis similarity, and a similarity profile test (SIMPROF) was calculated [44]. Due to the loss of the 15 m winter platform, again only 50–250 m bioerosion traces were included. One-way analysis of similarity (ANOSIM) was used to determine the significance of any clustering of bioerosion traces in the MDS ordination. Two-way ANOSIM was used to test the effects of depth, exposure, and orientation on bioerosion traces.

Normality tests were carried out with SigmaPlot. PERMANOVA, MDS, and ANOSIM were calculated using the Primer+PERMANOVA software package [45, 46].

## Results

### Water temperature

**Fig 3:**
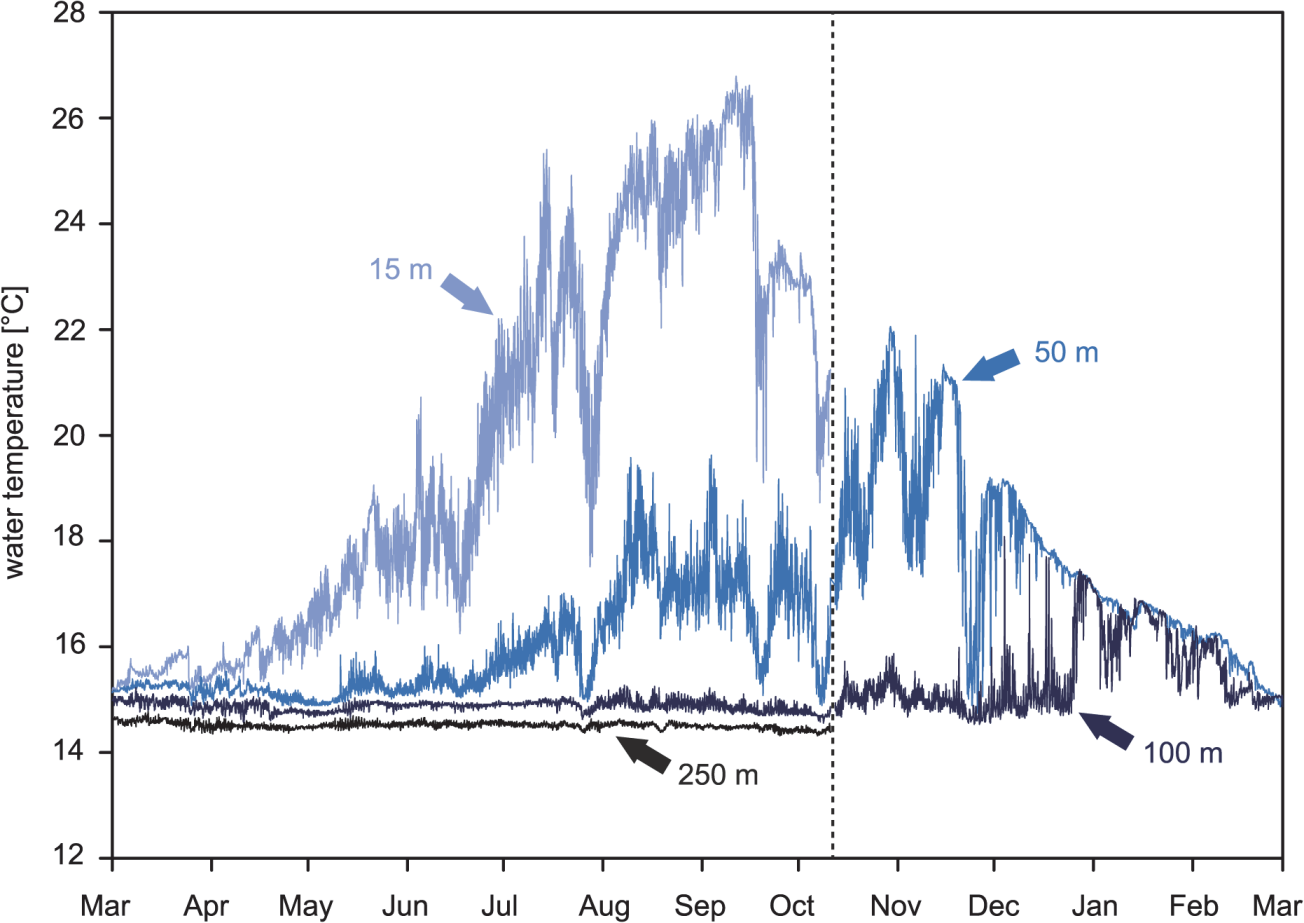
Water temperature measurement during the experiment from March 2008–2009. Dashed line marks the replacement from summer to winter platforms in October 2008. Due to platform and data loss, no water temperature is available for 15 and 250 m winter exposure, respectively.

Water temperatures along the bathymetric transect were subject of a distinct seasonal variability (Fig 3 and S1 Table). From April-November the water masses were highly stratified. Without the missing winter platform, the strongest fluctuation of 11°C was observed in 15 m water depth, with a minimum of 15°C in March-April, and a maximum of 26°C in September. Water masses in 50 m showed a variability of 7°C, with a minimum of 15°C until May, an increase up to 20°C until August, and a maximum of 22°C in November. In 100 m, temperatures were comparatively constant at 15°C until October, while from November a slight increase up to 18°C was recorded. Although the winter period temperature data is missing at the 250 m site, the temperature trend at this depth suggests a constant temperature of 14.5°C for the entire period of this study.

### Carbonate budget

#### Bioerosion rates

**Fig 4:**
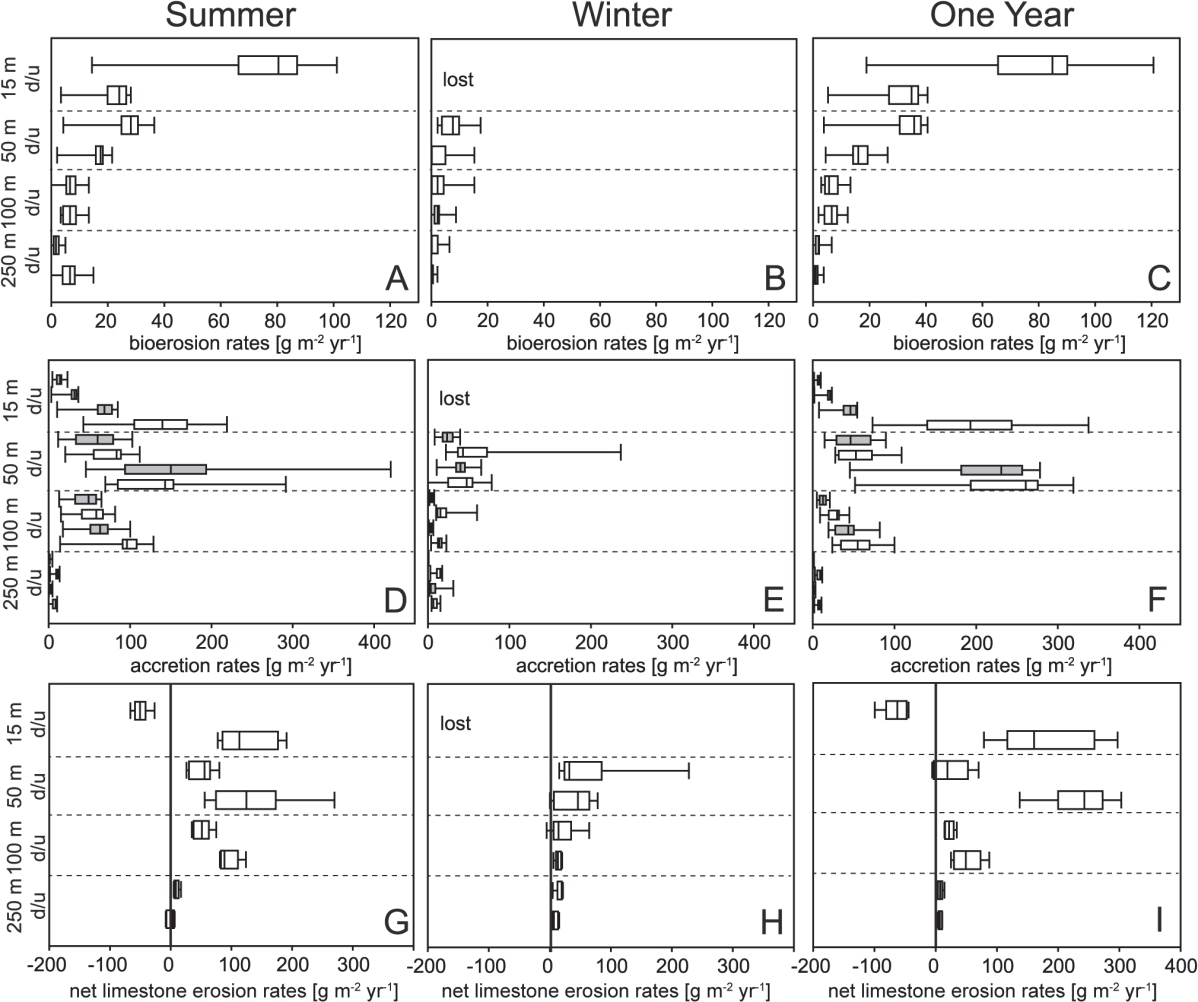
Box plots of bioerosion (A-C), accretion (D-F), and net limestone erosion rates (G-I) in up-facing (u) and down-facing (d) substrates from summer (A, D, G), winter (B, E, H), and one year exposure (C, F, I). Accretion is given for limestone (white) and PVC substrates (grey).

Highest bioerosion rates were measured in 15 m up-facing substrates during summer and one year exposure (mean 83 g m^-2^ yr^-1^), showing a distinct decrease towards deeper water (Fig 4A–4C and S2 Table). These values were about three-fold higher than in the 15 m down-facing substrates (mean 29 g m^-2^ yr^-1^). Three-way PERMANOVA of 50–250 m bioerosion rates revealed significant effects of depth and exposure (Table 1). Highest values of summer and one year bioerosion rates were reached in up-facing 50 m substrates (mean 32 g m^-2^ yr^-1^), while during the winter the bioerosion rates were distinctly lower (mean 8 g m^-2^ yr^-1^). In general, lowest values were measured in 250 m.

**Table 1:** Results of three-way permutational analysis of variance (PERMANOVA) testing effects of water depth, seasonal exposure, and substrate orientation on bioerosion rates from 50–250 m (statistically significant values are marked in bold).

Source	df	SS	MS	Pseudo-F	P (perm)
Depth	2	60.834	30.417	12.922	**0.009**
Exposure	2	39.932	19.966	8.482	**0.008**
Orientation	1	2.536	2.536	1.077	0.392
Depth x Exposure	4	25.400	6.350	2.698	**0.031**
Depth x Orientation	2	9.130	4.565	1.939	0.188
Exposure x Orientation	2	5.660	2.830	1.202	0.351
Residual	4	9.416	2.354		
Total	17	152.910			

#### Accretion rates

In contrast to bioerosion, accretion rates on up-facing substrates in shallow water were low, but peaked on down-facing substrates in 15 and 50 m water depth (Fig 4D–4F and S2 Table). Highest rates were observed on down-facing limestone and PVC substrates deployed in 50 m for one year (mean 241 g m^-2^ yr^-1^), and secondarily on 15 m limestone substrates (mean 213 g m^-2^ yr^-1^). During summer, carbonate production on those substrates was considerably lower (ca. 150–162 g m^-2^ yr^-1^). On 15 m down-facing substrates exposed for one year, the largest difference with regard to substrate composition was found with about five-fold higher carbonate accretion rates on PVC than on limestone substrates (mean 213 and 46 g m^-2^ yr^-1^). Four-way PERMANOVA of 50–250 m accretion rates revealed significant effects of depth, exposure, orientation, and substrate composition (Table 2). During summer and one year exposure, differences among carbonate accretion produced in 50 and 100 m were distinctly higher than during the winter. From 50–250 m, largest variability with regard to substrate orientation was observed on 50 m substrates during one year where about four-fold more carbonate was produced on down-facing than on up- facing substrates (mean 241 and 56 g m^-2^ yr^-1^). In 250 m water depth almost no carbonate was produced.

**Table 2:** Results of four-way permutational analysis of variance (PERMANOVA) testing effects of water depth, seasonal exposure, substrate orientation, and substrate type on accretion rates from 50–250 m (statistically significant values are marked in bold).

Source	df	SS	MS	Pseudo-F	P (perm)
Depth	2	271.940	135.970	75.014	**0.002**
Exposure	2	36.752	18.376	10.138	**0.006**
Orientation	1	12.062	12.062	6.655	**0.025**
Substrate	1	30.903	30.903	17.049	**0.007**
Depth x Exposure	4	36.875	9.219	5.086	**0.031**
Depth x Orientation	2	6.245	3.122	1.723	0.215
Depth x Substrate	2	18.664	9.332	5.148	**0.020**
Exposure x Orientation	2	6.647	3.324	1.834	0.212
Exposure x Substrate	2	4.113	2.057	1.135	0.424
Orientation x Substrate	1	7.466	7.466	4.119	0.061
Depth x Exposure x Orientation	4	12.365	3.091	1.705	0.191
Depth x Exposure x Substrate	4	10.952	2.738	1.510	0.244
Depth x Orientation x Substrate	2	4.589	2.294	1.266	0.356
Exposure x Orientation x Substrate	2	5.477	2.739	1.511	0.278
Residual	4	7.250	1.813		
Total	35	472.300			

#### Net limestone erosion rates

Calculated from the difference of accretion and bioerosion rates, net limestone erosion rates revealed a net loss of carbonate on 15 m up-facing substrates during summer and one year exposure (Fig 4G–4I and S2 Table). For all other substrates a net gain of carbonate was recorded. Highest net production of carbonate was recorded in 50 m down-facing substrates during one year of exposure (mean 235 g m^-2^ yr^-1^). Highest net loss of carbonate was measured in 15 m up-facing substrates during one year exposure (mean -65 g m^-2^ yr^-1^). Three-way PERMANOVA showed that net limestone erosion rates in 50–250 m were significantly affected by water depth (Table 3). For exposure and orientation no effect was found.

**Table 3:** Results of three-way permutational analysis of variance (PERMANOVA) testing effects of water depth, seasonal exposure, and substrate orientation on net limestone erosion rates from 50–250 m (statistically significant values are marked in bold).

Source	df	SS	MS	Pseudo-F	P (perm)
Depth	2	7.937	3.968	6.688	**0.033**
Exposure	2	1.202	0.601	1.013	0.435
Orientation	1	1.017	1.017	1.713	0.249
Depth x Exposure	4	2.518	0.630	1.061	0.470
Depth x Orientation	2	2.075	1.038	1.749	0.223
Exposure x Orientation	2	1.882	0.941	1.586	0.259
Residual	4	2.373	0.593		
Total	17	19.004			

### Bioerosion traces

**Fig 5:**
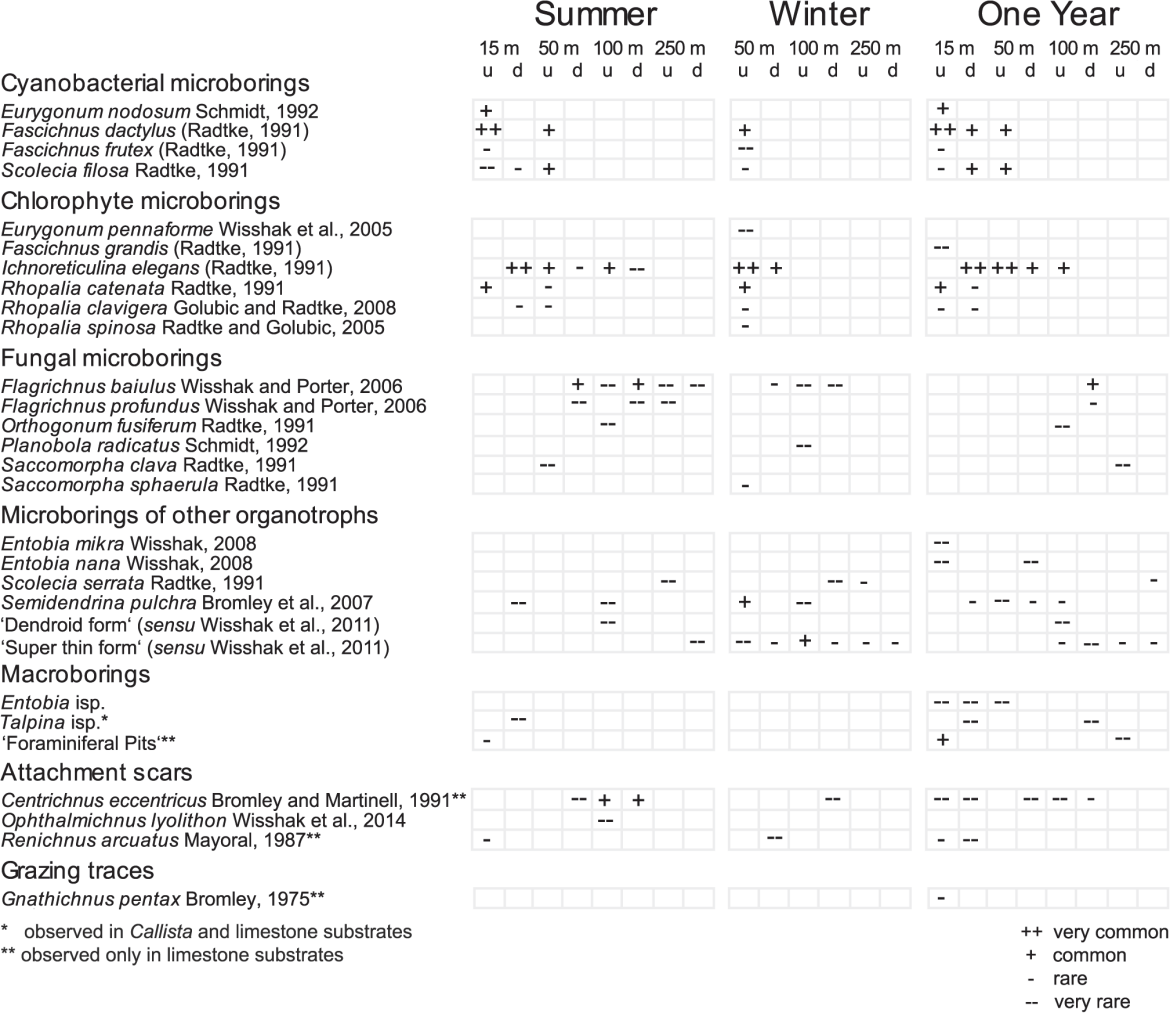
The inventory of bioerosion traces for the various water depths, substrate orientations (u = up-facing, d = down-facing), and exposure (summer, winter, and one year), categorised by trace types.

**Fig 6:**
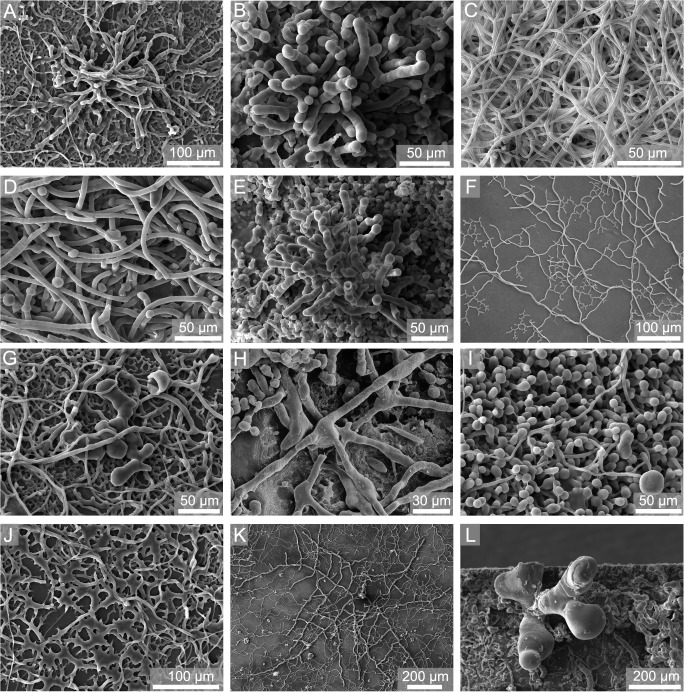
SEM images of epoxy-resin casts taken from *Callista* shells showing cyanobacterial (A-E) and chlorophyte (F-L) microborings. (A-B) *Fascichnus dactylus*, 50 m up, winter and 15 m up, one year, (C) *Scolecia filosa*, 15 m up, one year, (D) *Eurygonum nodosum*, 15 m up, one year, (E) *Fascichnus frutex*, 15 m up, one year, (F-G) *Ichnoreticulina elegans*, 100 m up, summer and 50 m up, winter, (H) *Rhopalia catenata*, 15 m up, summer, (I) *Rhopalia clavigera*, 50 m up, winter, (J) *Rhopalia spinosa*, 50 m up, winter, (K) *Eurygonum pennaforme*, 50 up, winter, (L) *Fascichnus grandis*, 15 m up, one year.

**Fig 7:**
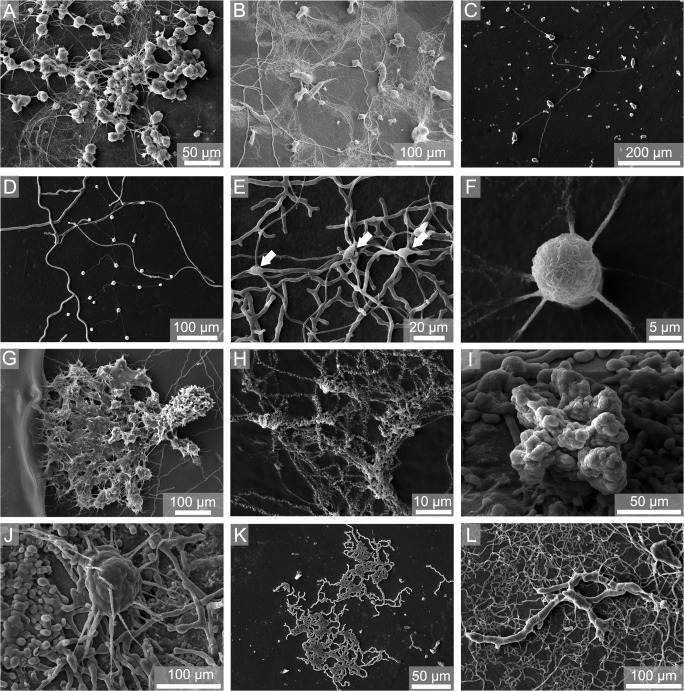
SEM images of epoxy-resin casts taken from *Callista* shells showing fungal microborings (A-F) and traces of other organotrophs (G-L). (A) *Flagrichnus baiulus*, 50 m down, summer, (B) *Flagrichnus profundus*, 50 m down, summer, (C) *Saccomorpha clava*, 250 m up, one year, (D) *Saccomorpha sphaerula*, 50 m up, winter, (E) *Orthogonum fusiferum* (arrows mark swellings), 100 m up, one year, (F) *Planobola radicatus*, 100 m up, winter, (G) *Semidendrina pulchra*, 50 m down, winter, (H) ‘Super thin form’, 250 m down, winter, (I) *Entobia mikra*, 15 m up, one year, (J) *Entobia nana*, 15 m up, one year, (K) *Scolecia serrata*, 250 m up, winter, (L) ‘Dendroid form 1’, 100 m up, one year.

**Fig 8:**
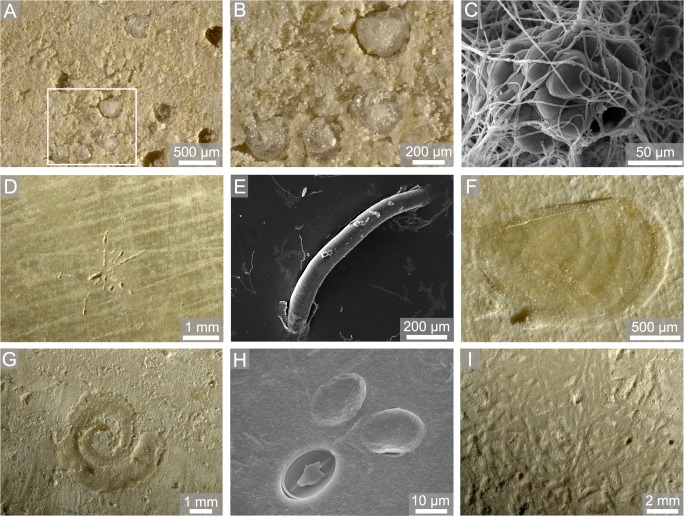
Light microscopic (A-B, D, F-G, I) and SEM images (C, E, H) of limestone blocks and *Callista* shells showing macroborings (A-E), attachment scars (F-H), and grazing traces (I). (A-B) ‘Foraminiferal Pits’, 15 m up, one year, (C) *Entobia* isp., 15 m down, one year, (D-E) *Talpina* isp., 50 m down, winter and summer, (F) *Centrichnus eccentricus*, 100 m down, summer, (G) *Renichnus arcuatus*, 15 m up, one year, (H) *Ophthalmichnus lyolithon*, 100 m up, summer, (I) *Gnathichnus pentax*, 15 m up, one year.

**Fig 9:**
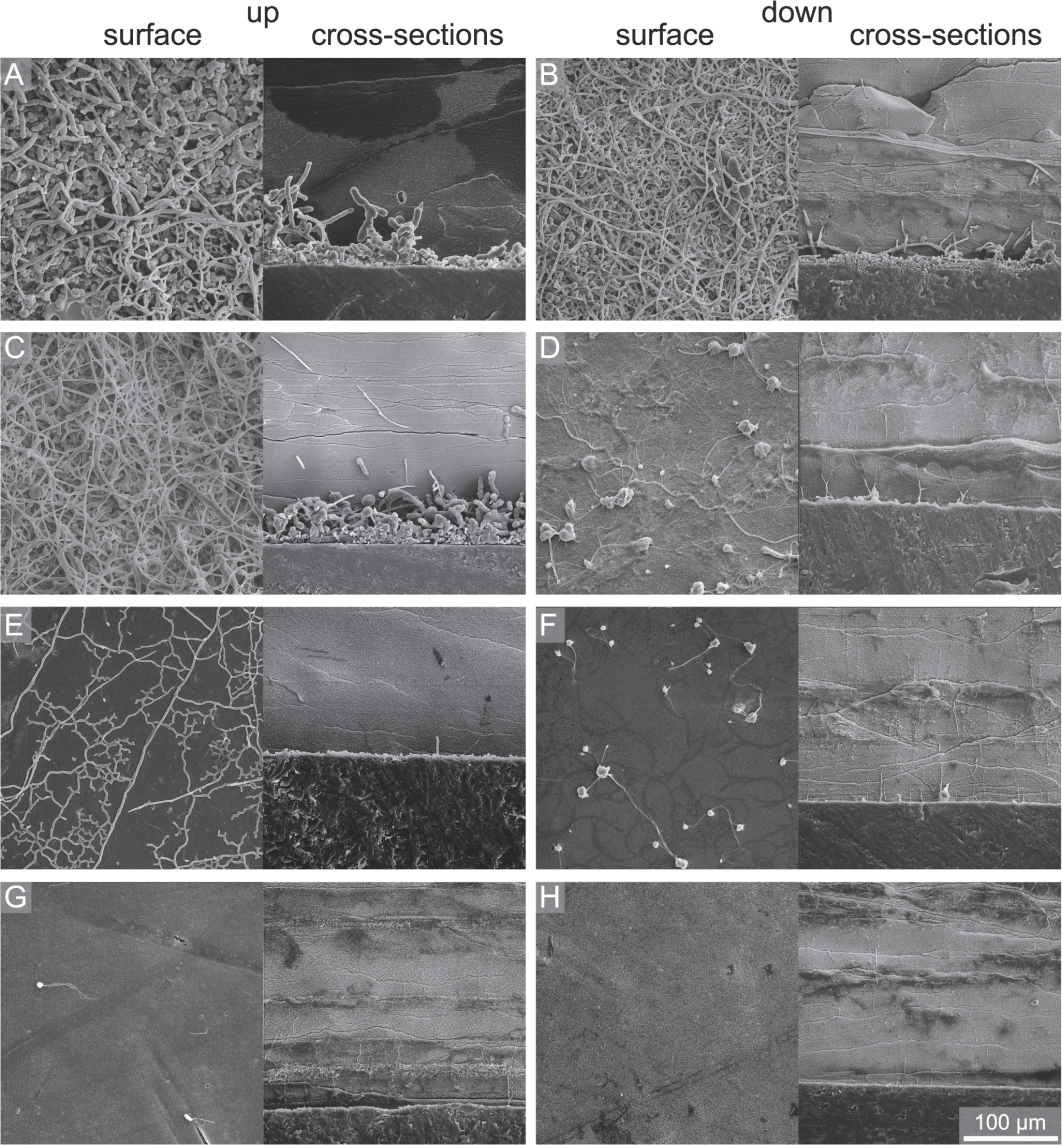
SEM images of summer samples of surface and cross-sections showing the degree of bioerosion with water depth and substrate orientation. (A) 15 m up, (B) 15 m down, (C) 50 m up, (D) 50 m down, (E) 100 m up, (F) 100 m down, (G) 250 m up, and (H) 250 m down.

**Fig 10:**
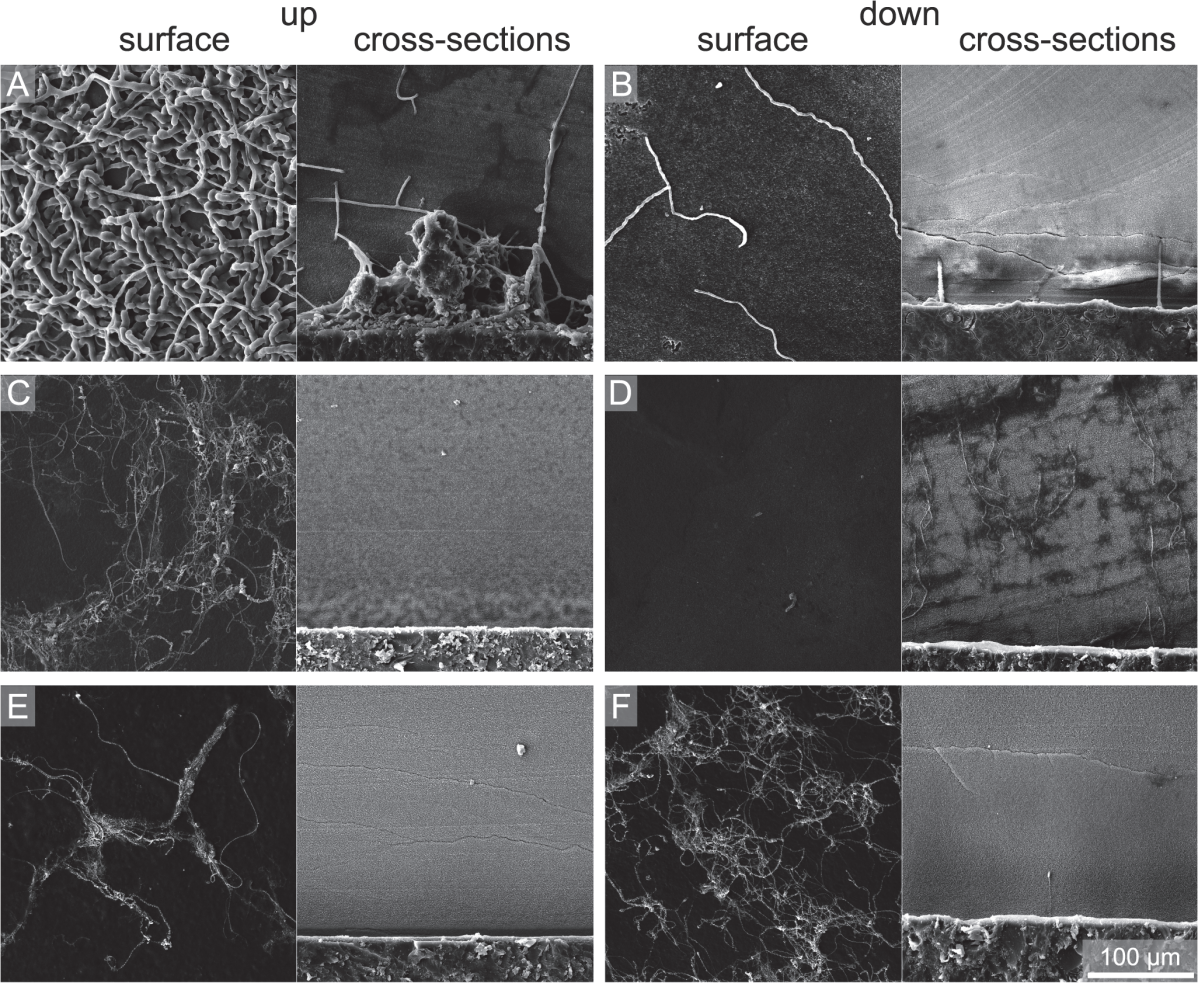
SEM images of winter samples of surface and cross-sections showing the degree of bioerosion with water depth and substrate orientation. (A) 50 m up, (B) 50 m down, (C) 100 m up, (D) 100 m down, (E) 250 m up, and (F) 250 m down.

**Fig 11:**
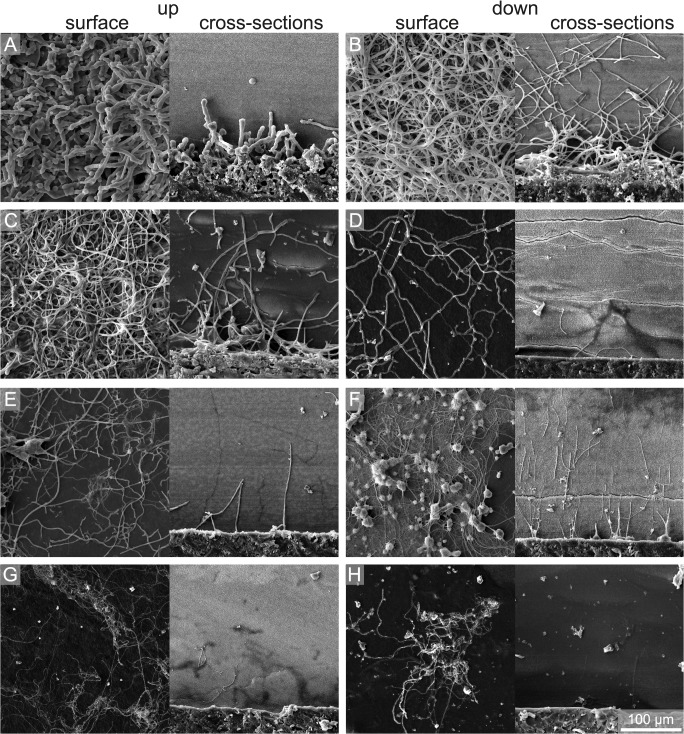
SEM images of one year samples of surface and cross-sections showing the degree of bioerosion with water depth and substrate orientation. (A) 15 m up, (B) 15 m down, (C) 50 m up, (D) 50 m down, (E) 100 m up, (F) 100 m down, (G) 250 m up, and (H) 250 m down.

In *Callista* shells and limestone plates a total of 29 different bioerosion traces was observed (Fig 5). These traces were attributed to 4 cyanobacterial microborings (Fig 6A–6E), 6 chlorophyte microborings (Fig 6F–6L), 6 fungal microborings (Fig 7A–7F), 6 microborings of other organotrophs (Fig 7G–7L), 3 macroborings (Fig 8A–8E), 3 attachment scars (Fig 8F–8H), and 1 grazing trace (Fig 8I). SEM overviews and cross-sections on the same scale are illustrated for summer (Fig 9), winter (Fig 10), and one year exposure (Fig 11).

The highest diversity of bioerosion traces was observed in 15 m up-facing substrates during one year of exposure, the lowest in substrates from 250 m water depth. During summer and one year exposure, up-facing substrates in 15 m were strongly colonised by cyanobacterial and chlorophyte microborings. Common were the cyanobacterial microborings *Fascichnus dactylus* and *Eurygonum nodosum* as well as the chlorophyte microboring *Rhopalia catenata*. Penetration depth was 15–100 μm. In 15 m down-facing substrates, cyanobacterial microborings were still common, but chlorophyte microborings became more abundant. Frequently galleries of the chlorophyte trace *Ichnoreticulina elegans* were observed. Mean boring depth was 10 μm with only few galleries surpassing 50 μm.

**Fig 12:**
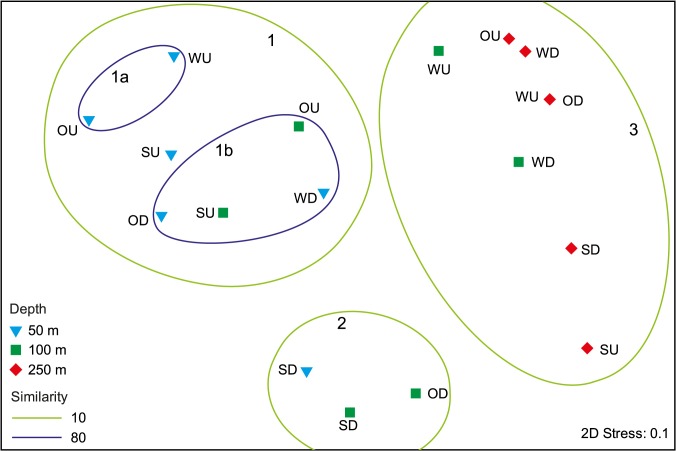
Multidimensional scaling plot (MDS) comparing summer, winter, and one year bioerosion traces observed in up- and down-facing substrates in 50–250 m water depth. Three main clusters (1, 2, 3) and two subclusters (1a, 1b) are separated with a Bray-Curtis-Similarity of 10 and 80, respectively. See S2 Fig for underlying cluster analysis (S = summer, W = winter, O = one year; U = up-facing, D = down-facing).

Multivariate analysis of bioerosion traces from 50–250 m separated three significantly different main clusters (ANOSIM R = 0.875, p = 0.001) and two subclusters (ANOSIM R = 1, p = 0.001) (Fig 12; see S2 Fig for underlying cluster analysis). Two-way ANOSIM showed that the occurrence of bioerosion traces was significantly affected by water depth and secondarily by orientation (Table 4).

**Table 4:** Results of two-way permutational analysis of similarity (ANOSIM) testing effects of water depth, seasonal exposure, and substrate orientation on bioerosion traces from 50–250 m (statistically significant values are marked in bold).

Comparison	Factor	R	p
Depth x Orientation	Depth	0.500	**0.002**
	Orientation	0.302	**0.046**
Depth x Exposure	Depth	0.472	**0.011**
	Exposure	-0.019	0.515
Orientation x Exposure	Orientation	-0.099	0.651
	Exposure	0.033	0.415

Cluster 1 contained bioerosion traces from up-facing 50 m summer, winter, and one year substrates, down-facing 50 m winter and one year substrates, and up-facing 100 m summer and one year substrates. In these substrates cyanobacterial and chlorophyte microborings dominated. The subclusters 1a and 1b further revealed a significant difference among bioerosion traces developed in 50 m up-facing substrates during winter and one year exposure, and those developed in 50 m down-facing winter and one year, and 100 m up-facing summer and one year substrates. Common traces in both subclusters were the chlorophyte microborings *I*. *elegans*. The major difference among these subclusters was the common occurrence of cyanobacterial microborings *F*. *dactylus* and *S*. *filosa* in subcluster 1a, while in subcluster 1b cyanobacteria were absent. Up-facing 50 m summer substrates differed from those in subcluster 1a and 1b showing commonly specimens of *F*. *dactylus* and *S*. *filosa*, but less *I*. *elegans*. Secondarily in 50 m up-facing winter substrates common specimens of the foraminiferan trace *Semidendrina pulchra* were observed. In 100 m up-facing winter substrates filaments of the ‘super thin form’ were common. Mean boring depth in 50 m up-facing substrates was still about 25 μm with few galleries surpassing 150 μm. In all other substrates the boring intensity was very low.

Cluster 2 contained bioerosion traces from 50 m and 100 m down-facing summer substrates and 100 m down-facing one year substrates. Typical were various specimens of the fungal microborings *Flagrichnus baiulus*. Cyanobacterial microborings were absent. With respect to chlorophyte microborings, only few galleries of *I*. *elegans* were detected. The boring intensity was very low.

Cluster 3 included bioerosion traces from 100 m up- and down-facing winter and all 250 m substrates. Solely fungal microborings and those of other organotrophs were observed. Cyanobacterial and chlorophyte microborings were absent. Borings were scarce and represented by galleries of fungal microborings *F*. *baiulus* and *F*. *profundus*, and the organotroph traces *Scolecia serrata* and ‘super thin form’. The boring intensity again was very low.

Excluded from statistical analyses, some conspicuous bioerosion traces were observed on limestone substrates. In 15 m up-facing one year plates, various small ‘foraminiferal pits’ were found, in which commonly tests of foraminiferans were preserved *in situ*. On 100 m up- and down-facing summer limestone plates, commonly the anomiid bivalve attachment scar *Centrichnus eccentricus* occurred. The echinoid grazing trace *Gnathichnus pentax* and initial sponge boring traces *Entobia mikra*, *E*. *nana*, and *Entobia* isp. were first observed during one year exposure.

## Discussion

### Effect of water depth

Our settlement experiment demonstrates that carbonate bioerosion and accretion in the Ionian Sea strongly vary with water depth. The comparison of bioerosion rates and traces showed that the highest bioerosion activity occurs in shallow water being caused by the boring activity of cyanobacteria and chlorophytes, while towards the fungal and organotrophic euendolith-dominated deeper water the bioerosion intensity strongly decreases (Figs 4 and 5). This general bathymetrical pattern is in good agreement with previous settlement experiments conducted at the Great Barrier Reef [15] and in the Azores Archipelago [16]. The restriction of phototrophic cyanobacteria and chlorophytes to shallow water originates from their specific low-light tolerance limit and light optimum, while fungi and organotrophic euendoliths are light independent and able to thrive also in aphotic depths [3, 47–50]. Microendolithic ichnocoenoses have long been identified to be a strong tool for judging light availability and hence relative bathymetry in both modern and ancient environmental settings [23]. According to the set of bathymetric index ichnocoenoses that was developed in order to reconstruct photic conditions in palaeoenvironments [15, 19, 20, 23], the dominance of the microboring trace index ichnotaxa *F*. *dactylus* and *R*. *catenata* in 15 m corresponds to typical shallow euphotic zone III ichnocoenoses from shallow subtidal ranges. In substrates deployed in 50 m water depth the common occurrence of *F*. *dactylus*, *S*. *filosa*, *I*. *elegans*, and few *S*. *clava* microborings reflects a transition between shallow euphotic zone III to deep euphotic ichnocoenoses. In 100 m the very common index ichnotaxon *I*. *elegans* and fungal microborings form a typical dysphotic ichnocoenosis, albeit *S*. *clava* was not observed. The exclusive occurrence of organotrophic microborings in 250 m substrates in the aphotic zone corresponds to typical aphotic ichnocoenoses although the index ichnotaxon *S*. *clava* was rare and *O*. *lineare* was not found. The low abundance and absence of these ichnotaxa is, however, not surprising, as they are known from other experiments to show only initial borings even after 1 and 2 years of exposure [17, 18].

In comparison to previous experiments from the Mediterranean Sea, the distribution of cyanobacterial and chlorophyte microborings from our experiment corresponds well with microbial endoliths observed during two colonisation experiments in the region of Marseilles in the Western Mediterranean Sea. Along a bathymetric transect from the supralittoral down to 190 m [30], the occurrence of the endolithic cyanobacteria *Mastigocoleus testarum*, *Hyella caespitosa*, and *Plectonema terebrans* down to 30, 60, and 80 m depth is in good agreement with their corresponding traces *E*. *nodosum*, *F*. *dactylus*, and *S*. *filosa* reported here from 15 and 50 m. The endolithic chlorophytes *Eugomontia sacculata*, *Phaeophila dendroides*, and *Ostreobium quekettii* occurred down to 80 and 100 m depth, which also corresponds well to the traces *R*. *clavigera*, *R*. *catenata*, and *I*. *elegans* we found down to 50 and 100 m. In a further colonisation experiment on rhodolith concretions in 10–60 m water depth [31], *H*. *caespitosa* was restricted to 10 m, *P*. *tenebrans* and *M*. *testarum* were found down to 20 m, and solely *O*. *quekettii* reached down to 60 m. The comparison of the bathymetric distribution of those microendoliths suggests a certain resemblance of the photic zonation in the Eastern and Western Mediterranean Sea.

A detailed description of calcareous epiliths was beyond the scope of our study and detailed information on carbonate production in the Eastern Mediterranean Sea is not available. A comparison with carbonate production on the Balearic Platform in the Western Mediterranean Sea [51], where large amounts of carbonate are produced in the bryozoan and red algae facies of the middle ramp-environment (ca. 30–90 m water depth), suggests that highest carbonate accretion rates in 50 m depth found in our study might likewise be produced by such low-light adapted epiliths.

In total, the calculation of net limestone erosion rates from the difference of carbonate bioerosion and accretion demonstrates the enormous effect of bioerosion on the carbonate budget of the Ionian Sea. While in deeper water (50–250 m) the net loss of carbonate by bioerosion is compensated by carbonate production, bioerosion rates exceed accretion in shallow water (15 m). In a similar settlement experiment along a more extended bathymetric transect in the Azores [16], highest mean bioerosion rates (456 g m^-2^ yr^-1^) were measured in the intertidal zone, whereas limestone accretion rates (7 g m^-2^ yr^-1^) were low (i.e. net limestone erosion rate of -449 g m^-2^ yr^-1^). This indicates that we might also expect increasing net loss of carbonate towards and in the intertidal zone in the Ionian Sea.

### Effect of seasonal exposure

The comparison of summer and winter platforms revealed a distinct seasonal effect on bioerosion and accretion. In 50 m, bioerosion rates of cyanobacteria and chlorophytes were distinctly higher during summer than during winter deployment (Fig 4). With regard to bioerosion traces, the absence of *I*. *elegans* borings in 100 m winter substrates indicates a shift in light supply from a dysphotic ichnocoenosis during summer to an aphotic ichnocoenosis during winter (Fig 5). These observations suggest a distinct seasonal variability in phototrophic microbioerosion, which is likely related to the reduction of absolute light availability during winter due to fewer hours of daylight and lower angles of irradiance. Both factors, however, do not alter the photic zonation, which is based on relative light levels (expressed in percentage of surface irradiance). Seasonal shifts of the photic zonation, in contrast, could originate from seasonal fluctuation in water turbidity. Turbidity effects on bioerosion were described from reef environments in Jamaica, where different light penetration regimes revealed marked variations in the bathymetric range of sediment microboring communities [52]. Seasonal variations in the turbidity regime of the Eastern Mediterranean Sea could be associated with the observed seasonal variability of water masses (Fig 3). Mean winter temperatures of 15°C agree with values reported for Modified Atlantic Water between 30–200 m in the Ionian Sea during winter [37]. Also the strong increase of water temperatures in 15 m is a typical phenomenon in the Ionian Sea where Ionian Surface Water clearly differs from the underlying Modified Atlantic Water due to surface heating [37]. In general, summer stratification conditions are considered to cause a depletion of particles and dissolved nutrients and a reduction of primary production in surface water in the Mediterranean Sea [34]. During winter, in contrast, the euphotic zone is enriched in nutrients by vertical mixing and plankton blooms resulting in higher water turbidity and could thus cause a shift of the photic zonation towards shallower water.

In addition to this indirect effect of water temperature on phototrophic bioerosion, higher water temperatures during summer may also directly increase the activity of phototrophic endoliths. Many phytoplankton cellular processes are temperature dependent and especially cyanobacterial growth rates are considered to be favoured by increasing water temperatures [53]. During the experiment highest temperatures of about 26°C were measured during September in 15 m water depth, where mostly cyanobacterial microborings were observed. In contrast to light, many euendoliths in modern seas are eurythermal and have a cosmopolitan distribution, and the evaluation of microborings as a palaeotemperature and/or palaeolatitude indicator is still in its infancy [23]. Optimal growth rates as well as lower and upper temperature limits of phototrophs are likely to be species specific and not presented for phototrophic microendoliths. Nevertheless, we suspect that in addition to the indirect effects of higher light availability and water transparency during summer stratification, water temperature might also directly enhance phototrophic microbioerosion during hot summer temperatures.

The difference among summer and winter bioerosion rates in 100 m depth was small. However, we observed a distinct seasonal difference in the abundance of the probable thraustochytrid fungal microboring *F*. *baiulus*, which was found in large quantities during summer. In contrast to cyanobacteria and chlorophytes, marine fungi are organotrophs that biodegrade calcareous shells in order to exploit mineralised organic matter and are independent of light supply [22]. Previously *Flagrichnus* borings were only described from non-tropical settings and were therefore suggested as an indicator for low (palaeo)temperatures [54]. The presence of *F*. *baiulus* during summer could be explained by the observation that substrates deployed in 50 and 100 m were exposed to comparatively low temperatures of 15–19°C, while during winter a distinct increase up to 22°C in 50 m and 17°C in 100 m was observed. A similar seasonal pattern was observed for thraustochytrid fungi in Mediterranean sandy habitats, where highest fungal densities were recorded in late spring and decreased during autumn-winter [55]. But the latter authors concluded that besides temperature other environmental factors such as particulate and/or dissolved organic matter could have contributed to the seasonal pattern they observed.

Another microboring that is considered to be indicative of cold-water conditions is the probable endolithic foraminiferan trace *S*. *pulchra* [56]. In contrast to *F*. *baiulus*, this trace was found during our experiment mostly in 50 m winter substrates, where a distinct increase of water temperature up to 22°C was recognised. For the suspension feeding foraminiferans, enhanced nutrient availability during winter could be related with higher amounts of inorganic nutrients during the winter plankton blooming period. However, we assume that similar to thraustochytrid fungi the occurrence of foraminifera is likely dependent on the interaction of several environmental parameters.

The minor difference between summer and one year bioerosion rates and microbioerosion traces suggests that the main settlement of microendoliths on one year substrates has taken place already during summer exposure. This corresponds to observations on coral reef ecosystems [6, 7], where microboring communities are considered to mature within 6 months of exposure. However, our results show that this applies only for summer exposure, while during the winter bioerosion rates the succession is strongly reduced. Due to practical reasons, summer exposure was about 50 days longer than winter exposure. Nevertheless, the observed differences in ichnocoenosis composition between the summer and winter period (e.g. *F*. *baiulus*) is unambiguous and very unlikely to have been caused by the minor difference in exposure period alone.

### Effect of substrate orientation

In comparison to water depth and exposure, only a minor effect of orientation was found. On the up-facing side in 15 m the dominance of the index ichnotaxa *F*. *dactylus* and *R*. *catenata* microboring traces corresponds to typical shallow euphotic zone III ichnocoenoses from shallow subtidal ranges (Fig 5). On the 15 m down-facing side, in contrast, the dominance of *I*. *elegans* and only few *R*. *clavigera* microborings represent a deep euphotic ichnocoenosis. In substrates deployed in 50 m water depth, the common occurrence of *F*. *dactylus*, *S*. *filosa*, *I*. *elegans*, and few *S*. *clava* microborings reflects deep euphotic ichnocoenoses on the up-facing side. On the down-facing side, the absence of *R*. *catenata*, but dominance of *I*. *elegans* and fungal traces are indicative for a dysphotic ichnocoenosis (although the index ichnotaxon *S*. *clava* was not observed). These findings suggest a distinct change in light supply among up- and down-facing substrates, which does not originate from a change in the photic zonation, but from shading effects on the down-facing side of the platform.

The inverse trend of higher bioerosion rates in up-facing substrates and higher accretion rates in down-facing substrates (Fig 4) was similarly observed in a settlement experiment in the Azores Archipelago [16, 40]. Their study found that in addition to lower bioerosion pressure the main factors for the preferential settlement of calcareous epiliths on down-facing substrates were shading, negative phototactic larval behaviour, lower predation pressure, lower competition with epiphytes, and lack of sediment smothering. However, dense epilithic colonisation on down-facing substrates could also hinder the settlement and activity of bioeroders [16].

## Conclusions

In conclusion, our study shows that bioerosion and accretion in the Ionian Sea were mostly affected by water depth and secondarily by seasonal exposure. Substrate orientation played a minor role. Elevated temperatures and high absolute light intensities during summer due to more hours of daylight and increased irradiance angles promoted phototrophic microbioerosion by endolithic cyanobacteria and chlorophytes in shallow water. Plankton blooms during winter mixing, in contrast, likely led to a seasonal variability in water turbidity and thus resulted in a shift of the photic zonation. Altogether, these variations revealed pronounced differences in the composition and bathymetric range of summer versus winter microbioerosion ichnocoenoses as well as linked rates of bioerosion. The observed patterns in overall bioeroder distribution and abundance were mirrored by the calculated carbonate budget with bioerosion rates exceeding carbonate production rates in shallow water and distinctly higher bioerosion rates at all depths during summer.

## Supporting Information

S1 Fig (A) Measurements of the photosynthetically active radiation (PAR; unit: μmol photons m^-2^ s^-1^), which were carried out during the MATER cruise in May 1996 in the Ionian Sea [38]. (B) Semi-logarithmic plot of corrected PAR values expressed as percent of the surface irradiance measured just below the water surface. Dashed line suggests a mean base of the euphotic zone (1% surface irradiance) in ca. 100 m of water depth and a base of the dysphotic zone (0.01% surface irradiance) in ca. 180 m. Graphs were displayed with Ocean Data View [39].
(TIF)

S2 Fig Group-average cluster analysis of bioerosion traces from 50–250 m water depth calculated based on Bray-Curtis-Similarity. Homogenous groups were coloured red during a similarity profile routine (SIMPROF) identifying three main clusters (green line = similarity of 10). The right cluster can be further subdivided in three subclusters (blue line = similarity of 80).
(TIF)

S1 Table Values of water temperature measurements during summer and winter deployment in 15, 50, 100, and 250 m. Due to the loss of a platform no data is available from the 15 m winter deployment. Due to data loss no values are available from the 250 m winter and all one year platforms.
(XLSX)

S2 Table Bioerosion, accretion, and net limestone erosion rates measured during the experiment. (XLSX)
