# Supplementary figures and images for: Effects of Water Depth, Seasonal Exposure, and Substrate Orientation on Microbial Bioerosion in the Ionian Sea (Eastern Mediterranean)
Source: PLoS One. 2015 Apr 20;10(4):e0126495. doi: 10.1371/journal.pone.0126495 (PMC4404344; doi:10.1371/journal.pone.0126495)

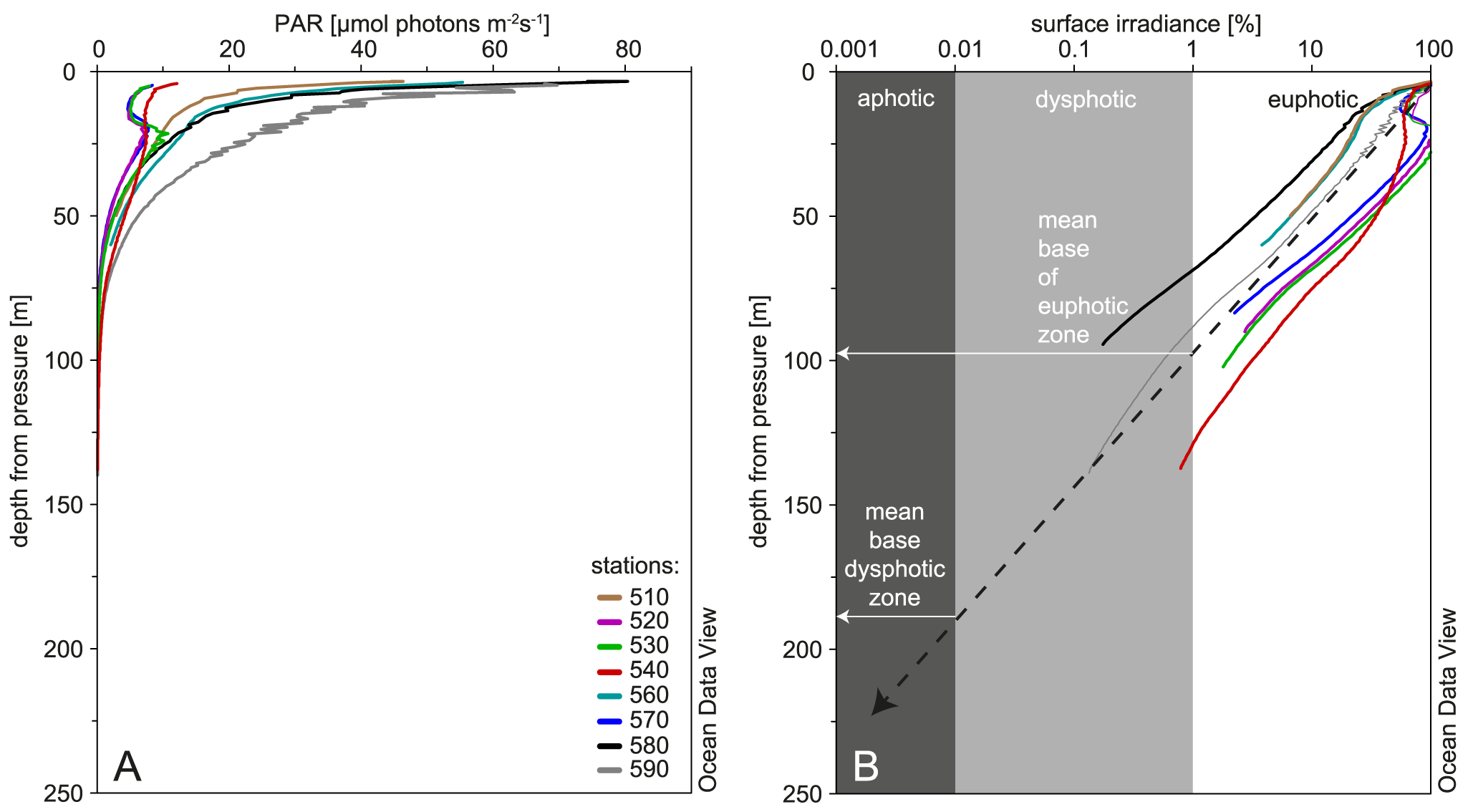

Supplement: S1 Fig — (B) Semi-logarithmic plot of corrected PAR values expressed as percent of the surface irradiance measured just below the water surface. Dashed line suggests a mean base of the euphotic zone (1% surface irradiance) in ca. 100 m of water depth and a base of the dysphotic zone (0.01% surface irradiance) in ca. 180 m. Graphs were displayed with Ocean Data View [39]. (TIF) [file pone.0126495.s001.tif]

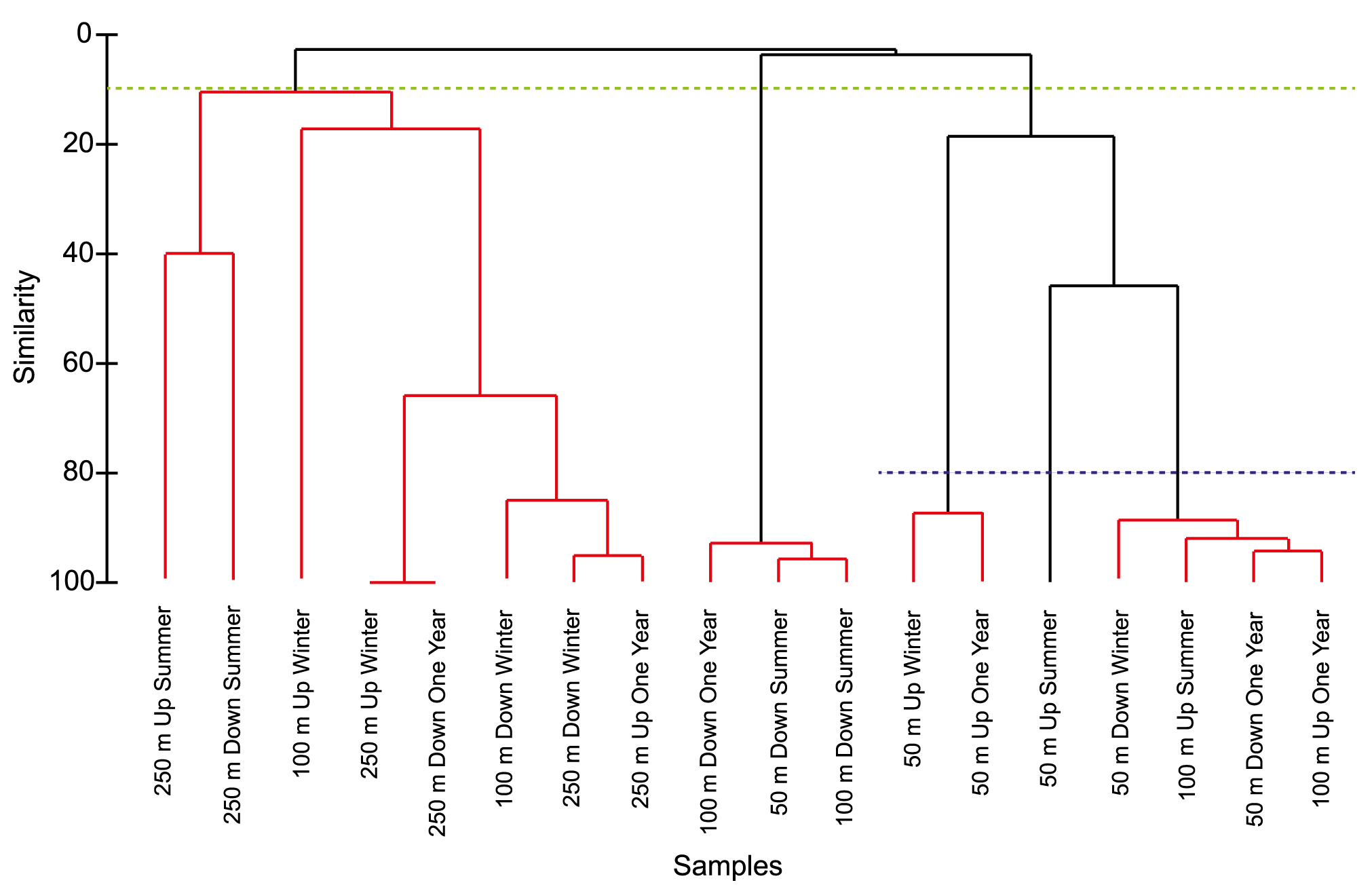

Supplement: S2 Fig — Homogenous groups were coloured red during a similarity profile routine (SIMPROF) identifying three main clusters (green line = similarity of 10). The right cluster can be further subdivided in three subclusters (blue line = similarity of 80). (TIF) [file pone.0126495.s002.tif]
